# Youth in the midst of escalated political violence: sense of coherence and hope among Jewish and Bedouin Arab adolescents

**DOI:** 10.1186/s13034-017-0178-z

**Published:** 2017-08-29

**Authors:** Sarah Abu-Kaf, Orna Braun-Lewensohn, Tehila Kalagy

**Affiliations:** Conflict Management and Resolution Program, Ben-Gurion University of the Negev, P. O. Box 653, 84105 Beer-Sheva, Israel

**Keywords:** Adolescents, Political violence, Sense of coherence, Hope, Bedouin Arab

## Abstract

**Background:**

During stressful events, individuals (particularly adolescents) from minority groups are often more vulnerable to distress. This claim will be examined in terms of coping resources and stress reactions to escalated political violence. This study aimed to compare coping resources and stress reactions among adolescents from two ethnic groups in southern Israel—Jews and Bedouin Arabs—during a period of escalated political violence (November 2012). The Bedouin Arab group is the ethnic minority of the sample and thus may be more at risk compared to the Jewish group.

**Methods:**

Data were gathered from 78 Jews and 91 Bedouin Arabs (14–18 years old) by using convenience sampling method. Adolescents were contacted via the Internet or in person by the research team and they completed self-report questionnaires including the Sense Of Coherence Scale (SOC), Hope Index, State Anxiety Inventory, and State Anger Inventory. After a preliminary χ^2^ analysis of the sample characteristics, three main sets of analyses were conducted including a two-way MANOVA, zero-order correlations between study variables, and hierarchical multiple regressions.

**Results:**

Bedouin Arab adolescents reported lower levels of SOC (*F*(1, 158) = 3.88, *p* = 0.04) and higher levels of individual and collective hope (*F*(1, 158) = 3.94, *p* = 0.03; *F*(1, 158) = 17.41, *p* = 0.001, respectively), as compared to Jewish adolescents. The Bedouin adolescents also reported higher levels of state anger *(F*(1, 158) = 5.58, *p* = 0.02). We identified cultural similarities related to the predictive power of coping resources (SOC and individual hope) in explaining state anger (β = −0.29, *p* = 0.001; β = −0.18, *p* = 0.045, respectively). However, cultural differences were found to affect the ability of SOC to predict state anxiety; SOC contributed significantly to state anxiety only among the Jewish adolescents (β = −0.45, *p* < 0.001).

**Conclusion:**

These results emphasize the significance of addressing cultural/ethnic factors in attempts to understand mental-health issues among youth during periods of escalated political violence.

## Background

Studies that have compared individuals from majority and minority groups during or following stressful events have found minorities to be more vulnerable to distress [1–3]. Moreover, adolescents have been found to be at risk for psychological distress and posttraumatic stress reaction following an incident of political violence [4]. The Bedouin Arabs in the Negev region of Israel number 230,000 and comprise 23% of the population of the Negev [5]. As an ethnic minority group, the Bedouin Arab population in Israel faces enormous difficulties in the social, cultural, political, and financial domains of everyday life [6]. This society is characterized by a young population (over 60% are under age 19) and high levels of poverty [6]. Bedouin Arab society also differs from Jewish Israeli society in terms of language, religion, and other cultural characteristics [7]. Bedouin Arab culture is highly collectivistic, patriarchal, and authoritarian [8] and differs from Jewish Israeli culture in terms of its emphasis on collectivistic values [9, 10].

Bedouin Arab society exhibits a high degree of power distance, which means each person has a place and there is no need for justification [9]. This cultural context accepts a hierarchical order in which inequalities based on gender and ages are common [11, 12]. We suggest that these values may affect the sense of power and levels of stress experienced by young individuals.

During November 2012, as hundreds of missiles were fired from Gaza at cities and other sites around Israel, an intensive military operation was directed at and inside the Gaza Strip. Those events of political violence may have imposed additional stress upon Bedouin Arab adolescents due to the confusing political situation with which they were faced. On the one hand, these adolescents felt angry with the Israeli leadership which directed the Israeli military to bomb Gaza. Some of these adolescents had close relatives in the Gaza Strip (Welfare Office, Rahat Municipality, personal communication, June 12, 2014). On the other hand, they themselves were living under the threat of missiles, which were falling on their city, Rahat.

The Jewish adolescents in our study belong to the majority culture in Israel (74.8%). Jewish Israeli society is characterized as more individualistic and less authoritarian than Bedouin Arab society and places a greater emphasis on separation, independence, personal development, and achievement [9, Welfare Office, Rahat Municipality, personal communication, June 12, 2014]. In comparison to Bedouin Arab society, Jewish Israeli society tends to be more modern and Western. The Jewish Israeli family is a nuclear system characterized by democratic family relations with relatively permissive parental control [8, 13]. During the escalated political violence, Jewish communities in southern Israel faced the same missile threat.

In this study, we compare coping resources and their associations with stress reactions among Bedouin and Jewish adolescents during this period of escalated political violence. The aim of this study was to explore the prevalence of coping resources and stress reactions among Bedouin Arab and Jewish adolescents, in order to evaluate differences in vulnerability to distress and resilience between these groups.

### The salutogenic model and sense of coherence

Researchers and theorists within virtually every subdiscipline of psychology have acknowledged the relevance of subjective construal and self-construal. While construal is a perception of the individual’s surroundings, self-construal is perception of one’s self [14]. The concept of construal can be seen in the works of many social psychologists including Kurt Lewin’s recognition of the importance of a subjective reality and its effect on one’s personal significance [15], and Brunswik’s theories of social perception [16]. Construal was once viewed as an obstruction in one’s perception of the world, but that understanding has evolved and construal is now viewed as a mechanism that can explain how or why individuals think the way they do [17].

Later, in the mid-1970s, a theory from medical sociology was developed and called the *salutogenic model*. This model suggests that life is full of stressful events and (similar to the construal concept) that the subjective meaning and the individual’s perception of an event have more important consequences than the event itself [18]. Thus, it would appear to be crucial to explore which resources lead an individual to perceive an event in a particular manner and to assign a particular subjective meaning to that event. Antonovsky [18] suggested two main concepts to provide a comprehensive answer to this question: generalized resistance resources (GRRs) and sense of coherence (SOC). GRRs include characteristics of the individual, group, or environment (subculture or society) that promote effective coping with stressful situations. Internal GRRs include cognitive and emotional resources and external GRRs include financial resources, living conditions, education, and social networks. The more GRRs one possesses the better one’s chances of overcoming a stressor [19]. According to Antonovsky [18], SOC strength is derived from GRRs.

Sense of coherence refers to an enduring attitude and measures how people view life, as well as how they identify, use, and reuse their GRRs to maintain and develop their health in the face of stressful situations. SOC has important implications for the ways in which individuals react to various kinds of stressful situations (for reviews of this topic, see [20, 21]).

Empirical research has shown that individualistic and collectivistic societies have different effects on individuals’ self-concepts and interpersonal relationships, as well as on their emotional and cognitive development [10, 15, 22]. Kagitcibasi [23] distinguished between collectivist and individualist societies, describing them as “the cultures of relatedness and separateness,” respectively. Collectivist cultures are characterized by a special concern with relationships and their maintenance. In collectivistic cultures that encourage a view of the self as interdependent, individuals are strongly motivated to adjust to and meet the expectations of socially meaningful others [16, 24]. Individualistic cultures, on the other hand, tend to encourage a view of the self as independent and in those cultures individuals are strongly motivated to confirm positive, self-defining attributes of the self, such as competence and efficacy [25]. Accordingly, it can be assumed that different cultural contexts may affect how people define themselves and view life, as well as which factors are considered to be resources and how individuals prefer to use and reuse their GRRs to maintain and develop their health in the face of stressful situations. Independent of collectivism and individualism, SOC is associated with lower levels of depression, neuroticism, and anxiety, as well as greater life satisfaction [21]. Based on the salutogenic approach, SOC is assumed to mediate relationships between exposure to political violence and stress reactions [26]. Individuals with a strong SOC will be less likely to feel threatened by events of war, such as missile attacks, and will be less emotionally vulnerable after having experienced such events [20, 27].

Several studies have explored SOC among individualistic majority and collectivistic minority groups around the world and the results of those studies have been inconsistent. While some collectivistic minority groups exhibit strong SOC that is similar to that of individualistic majority groups [28, 29], other collectivistic minority groups have been shown to exhibit weaker SOC than their individualistic majority counterparts (e.g., [1, 30]).

Studies of salutogenesis among adolescents have found that the relations between SOC and health or mental health among adolescents are similar to those observed among adults (e.g., [31, 32]). The better one’s health is perceived to be, the higher one’s SOC and, at the same time, the less severe one’s subjective health or mental-health complaints.

In addition to the collectivistic cultural orientation, the Bedouin Arab community in southern Israel is underprivileged in many areas (e.g., social, educational, political, and financial; [33]). Thus, Bedouin Arab adolescents have lower levels of GRRs (limited financial resources, harsh living conditions, lower levels of education) and SOC, as compared to their Jewish peers (e.g., [30]). Moreover, these adolescents belong to a society that is currently undergoing a rapid transition process.

### Hope

According to Staats [34], hope is “intrinsically a positive affective cognition in the subjective present” (p. 22). Hope consists of cognitive elements of visualization and expectation, as well as affective elements of feeling good about expected pleasant events or outcomes [35]. However, Staats placed more emphasis on the affective component.

Other researchers have offered different definitions of hope and emphasized different components of this construct. For example, Folkman [36] discussed hope from the vantage of stress and coping theory. Her main assumption was that hope is essential when we need to confront stressful situations, but is not always available. Moreover, hope can sustain coping, when the individual moves forward to deal with the demands of his or her new challenging reality. Previous research has recognized the importance of hope as a resource that has lasting effects on an individual’s ability to cope with stressful situations [36, 37]. Hope seems to be particularly important among adolescents, who are known to be vulnerable to depression, pessimism, and learned helplessness [37]. Hope has individualistic (hope for the self) and collectivistic (hope for the other) components [38].

As members of a highly collectivistic culture, defined by Schwartz and Bilsky [39] as a culture that prioritizes “in-group goals over personal goals” (p. 140), Bedouin individuals are motivated to wish for and promote the goals of others (the collective) before or at the expense of their own personal goals [9, 40, 41]. However, these youths may also be affected by Western individualistic values through rapid change process and those values would encourage them to wish for, expect to achieve, and promote their own personal goals [42].

A study that examined individual and collective hope among Israeli and Palestinian youth during periods of political violence reported similarly high levels of individual hope among the two groups. However, levels of collective hope were higher among Palestinian adolescents than among their Israeli counterparts [43]. The authors of that work attributed these results to cultural differences. To the best of our knowledge, no research has been conducted to identify the unique role of individual or collective hope in predicting reactions to stress.

### The role of demographic variables

Gender has been found to have a significant effect on stress reactions. The majority of studies reported in the literature confirm the importance of this gender effect, with girls generally expressing more distress and internalization of difficulties than boys. Boys, on the other hand, exhibit more externalization of problems and more risk-taking behavior [44, 45].

Age is considered to be a protective factor against stress. Several studies have found that younger children exhibit more severe psychopathology (e.g., somatic complaints, depression, and distress) in response to stress, as compared to older children and adolescents [46]. However, other studies focused on ongoing exposure to political violence reported no age effects [46, 47].

### Research hypotheses

This study was conducted during a military operation in the Gaza Strip known as Operation Pillar of Clouds during which southern Israel was under intensive missile attack. We examined how coping resources (i.e., SOC, individual hope, and collective hope) explain the stress reactions of state anger and state anxiety. The two stress reactions were analyzed separately.

In accordance with the research goals, the following research hypotheses were formulated:

1. Based on previous research, we expected that the Bedouin Arab adolescents would report lower levels of SOC than the Jewish adolescents. We expected to find higher levels of collective hope among the Bedouin Arab adolescents. In addition, we expected the Bedouin Arab adolescents to present higher levels of state anger than the Jewish adolescents, but similar levels of anxiety [30].
2. We expected to find negative associations between SOC or hope components and stress reactions (state anger and/or state anxiety). Stronger correlations were expected between SOC and stress reactions among Jewish adolescents; however, stronger correlations were expected between hope components and outcomes among Bedouin Arab adolescents [30, 43, 48, 49].
3. Based on previous research, we expected SOC to play a significant role in explaining stress reactions mainly among the Jewish group [48, 49]. However, we also expected that hope components would play a significant role in explaining stress reactions mainly among the Bedouin Arab group [30, 43].

## Methods

We employed a cross-sectional research design. The current study was conducted during November 2012. 169 participants were included by using convenience sampling method. During this period, schools were closed and people tended to stay at home. During the military operation, when adolescents were at home all day, the best way to connect with Jewish adolescents was via the Internet. Advertisements with the link to the questionnaires were published in Arabic and Hebrew via the services of the online panel company Midgam (http://www.midgampanel.com). Bedouin Arab adolescents were recruited from the city of Rahat, which was the only Bedouin community within the range of missile-fire. In the beginning of the period of escalated political violence, the research team tried to contact Arab adolescents and their families via the Internet. However, we received a very low response rate. The families and the adolescents were uncomfortable discussing their experiences with the stressful situation with strangers (online). Thus, we decided that the Bedouin Arab researcher/first author would contact them personally and emphasize the anonymity of this study. One hundred and ten Bedouin Arab adolescents and their parents were approached by the first author and two female research assistants and only 91 agreed to participate in the study (response rate of 83%). No inclusion or exclusion criteria were used with the exception of age (14–18 years). All participants were informed that the researcher was interested in their experience during the period of missile attacks, and it was emphasized that there are no right or wrong answers.

### Measures

#### Sense of coherence (SOC)

Sense of coherence [49] was measured using a series of 13 semantic differential items each rated on a 7-point Likert-type scale. High scores indicate a strong SOC. An account of the development of the SOC scale and its psychometric properties, showing it to be reliable and reasonably valid, appears in Antonovsky [19, 50]. The scale includes such items as “Doing the things you do every day is …” with answers ranging from 1 (*a source of pain and boredom*) to 7 (*a source of deep pleasure and satisfaction*). In the present study, the Cronbach’s alpha coefficients of reliability for this scale were 0.83 for the Jewish sample and 0.67 for the Bedouin Arab sample. Among the latter group, the reliability of this measure was improved to 0.72 when item 10 was deleted. That item included the statement: “There are many people, even those with strong characters, who sometimes feel miserable\poor.” This item was deleted only among the Bedouin Arab sample. Since Cronbach’s alpha coefficient of 0.67 is very low, this step was necessary in order to enable us to use the SOC scores in the different statistical analyses among the Bedouin Arab sample. An explanation of the difficulty with the specific item will be addressed later in the discussion.

#### Hope index (HI)

This index [38] represents the interaction of wishes and expectations and includes items regarding hopes for one’s self (individual hope) and hopes concerning others or broad global concerns (collective hope). Participants were asked to independently rate the extent to which they wished for a particular future occurrence and the extent to which they expected it to occur. Responses were rated on a scale of 0 (*not at all*) to 5 (*very much*). The multiplication of the wish value by the expect value generated the measure of hope. The Cronbach’s alpha coefficients of reliability for individual hope and collective hope ranged from 0.85 to 0.95 in both samples.

#### State anxiety and state anger

Adolescents’ anxiety was measured in terms of state anxiety and their anger was measured in terms of state anger [51, 52]. The Hebrew measure has been proven to be reliable, valid, and equivalent to the English State Anxiety and Anger Inventory [52]. The subscale of state anxiety consists of 11 items, which are scored on a 4-point scale. Mean scores for each subscale were used. These outcome measures are not clinical instruments thus there are no clinical cutoff levels. The closer the mean score is to 4 the higher the level of anxiety and anger experienced by the individual. The Cronbach’s alpha coefficients of reliability for state anxiety and state anger ranged from 0.75 to 0.90 in both samples.

#### Demographic questionnaire

This questionnaire included questions regarding participant gender, age, cultural group (i.e., Jewish or Arab), and socioeconomic status.

### Statistical analysis

The current study was analyzed using IBM SPSS Statistics 24 software. Prior to the main analysis, data were screened by analyzing frequencies and descriptive statistics such as means, standard deviations, and ranges. No outliers or out-of-scale points were found. A missing-values analysis was conducted, and because for some variables more than 5% of the data were missing at random, missing data were estimated using expectation–maximization methods. A preliminary χ^2^ analysis explored differences in sample characteristics between the two cultural groups. Then, three main sets of analyses were conducted. First, a two-way MANOVA with five dependent variables (DV) (SOC, individual hope, collective hope, state anger, and state anxiety) was used to explore the effects of cultural group, gender, and the interaction between culture and gender on the main study variables. Given the significance of the overall test, the univariate main effects were examined. Second, we computed zero-order correlations between coping resources and stress reactions among Bedouin Arab and Jewish students. Finally, we conducted hierarchical multiple regressions to answer our question concerning coping resources that may predict state anxiety and state anger in the different groups. Two hierarchical multiple regressions with interaction terms [53] were computed separately for each outcome variable (state anger and state anxiety). The predictors were: culture, age, SOC, individual hope, collective hope, and two-way interactions terms involving culture and SOC, culture and individual hope, and culture and collective hope.

### Ethical considerations

This study was approved by the Ethics Committee of the Conflict Management and Resolution Program of Ben-Gurion University of the Negev. Informed consent and consent for publication were obtained from participants (or their parent or legal guardian in the case of those under age 16). We emphasized that the participation was voluntary and anonymous, and that they were free to withdraw their participation for any reason and at any time during the questionnaire administration. Data were collected using Arabic and Hebrew versions of questionnaires (which were developed as part of previous research) during November 2012, as hundreds of missiles were fired on many locations in Israel and an intensive military operation was conducted on and inside the Gaza Strip.

## Results

### The study population

One hundred and sixty-nine teenagers living in southern Israel participated in this study: 78 Jews and 91 Bedouin Arabs. A preliminary analysis indicated nonsignificant age differences between the two samples. However, the Bedouin Arab sample included more females and religious adolescents, as well as lower parental educational level as compared to the Jewish sample (Table 1).

**Table 1:** Demographic characteristics: Differences between Bedouin Arab and Jewish adolescents

	Bedouin Arabs *N* = 91 (%)	Jews *N* = 78 (%)	*χ* ^2^	*p* value
Age, mean (*SD*)	16.48 (1.14)	16.77 (1.13)	1.63	*0.952*
Participant gender			32.46	*0.001*
Male	26.1	44.9		
Female	73.9	55.1		
Religiosity (Jewish/Bedouin Arab)				
Secular/not religious	2.2	51.3	37.55	*0.000*
Traditional/somewhat religious	34.4	23.1		
National religious/religious	63.4	20.5		
Ultra-orthodox/highly religious	0.0	5.1		
Income			1.44	*0.13*
Below average	64.9	55.1		
Average	19.3	23.6		
Above average	15.8	21.3		
*Parental education level*			*29.56*	*0.001*
*No formal education*	*6.4*	*19.8*		
*Elementary school*	*11.5*	*17.7*		
*High school*	*39.7*	*50.5*		
*Higher education*	*42.4*	*11.0*		

*SD* standard deviation

### Cultural and gender differences

A two-way MANOVA with five dependent variables (SOC, individual hope, collective hope, state anger, and state anxiety) revealed significant multivariate main effects of culture, participant gender, and the Culture × Gender interaction Given the significance of the overall test, the univariate main effects were examined and are reported in Table 2.

**Table 2:** Coping resources and stress reactions: means and standard deviations for each subgroup and effects of culture, gender, and culture*gender

Study variables	Bedouin Arab students			Jewish students	Culture	Gender	Culture*gender
	Female (*n* = 67)	Male (*n* = 24)	Female (*n* = 43)	Male (*n* = 35)			
	M (SD)	M (SD)	M (SD)	M (SD)	*F*(1, 158) (p value)	*F*(1, 158) (p value)	*F*(1, 158) (p value)
Coping resources							
SOC	3.73 (0.84)	4.07 (0.62)	4.26 (1.03)	4.51 (0.92)	3.88 (*0.04*)	2.33 (*0.08*)	2.24 (*0.09*)
Individual hope	18.39 (5.62)	15.33 (4.16)	15.26 (5.48)	14.20 (4.29)	3.94 (*0.03*)	3.5 (*0.06*)	0.21 (*0.56*)
Collective hope	16.07 (7.45)	14.36 (6.53)	9.01 (6.35)	9.36 (6.48)	17.41 (*0.001*)	0.22 (*0.13*)	1.3 (*0.24*)
Stress reactions							
Anxiety	2.45 (0.50)	2.36 (0.51)	2.53 (0.72)	1.97 (0.47)	0.55 (*0.32*)	7.90 (*0.009*)	4.11 (*0.03*)
Anger	2.02 (0.69)	2.03 (0.57)	1.91 (0.90)	1.46 (0.52)	5.58 (*0.02*)	2.35 (*0.08*)	2.65 (*0.07*)

*n* sample size, *M* mean, *SD* standard deviation

As compared to their Jewish counterparts, Bedouin Arab adolescents reported significantly higher levels of individual hope, collective hope, and state anger, as well as significantly lower levels of SOC. Gender differences were found only for state anxiety, with females reporting higher levels of state anxiety than male participants. Specifically, Jewish females reported higher levels of state anxiety than Jewish males. However, no effect of gender was noted in the Bedouin Arab sample (see Table 2).

### Association between coping resources and stress reactions among Bedouin Arab and Jewish students

Among the Jewish adolescents, there were negative moderate associations between SOC and stress reactions (anxiety and anger), as well as negative weak associations between individual hope and the two stress reactions. The higher the levels of SOC and individual hope of the adolescent, the lower the levels of state anger and state anxiety he\she reported. Among the Bedouin Arabs, there was negative weak association between SOC and anger; moreover, negative weak associations were noted between the two types of hope and anger, but not with anxiety (see Table 3).

**Table 3:** Zero-order correlations observed between the study variables

	1	2	3	4	5
1. SOC	*1*	*0.17* (*p* *=* *0.000*)	*0.12* (*ns.*)	*−0.14* (*ns.*)	*−0.23* (*p* *=* *0.03*)
2. Individual hope	*0.39* (*p* *=* *0.000*)	*1*	*0.71* (*p* *=* *0.000*)	*−0.15* (*ns.*)	*−0.21* (*p* *=* *0.04*)
3. Collective hope	*0.40* (*p* *=* *0.000*)	*0.58* (*p* *=* *0.000*)	*1*	*−0.14* (*ns.)*	*−0.23* (*p* *=* *0.03*)
4. Anxiety	*−0.49* (*p* *=* *0.000*)	*−0.23* (*p* *=* *0.032*)	*−0.15* (*ns.*)	*1*	*0.61* (*p* *=* *0.000*)
5. Anger	*−0.44* (*p* *=* *0.000*)	*−0.22* (*p* *=* *0.035*)	*−0.16* (*ns.*)	*0.69* (*p* *=* *0.000*)	*1*

Correlations above the diagonal are for the Bedouin Arab group (*n* = 91) and correlations below the diagonal are for the Jewish group (*n* = 78)

*ns.* non-significant

### Multiple regression analysis of factors predicting state anger

As shown in Table 4, overall, the regression model explained 20% of the explained variance in state anger. Culture significantly predicted state anger. Bedouin Arab culture predicted a higher level of state anger. Moreover, SOC and individual hope contributed to the explanation of this outcome. Higher levels of SOC and individual hope predicted lower levels of state anger, whereas collective hope and interaction terms did not predict the levels of state anger.

**Table 4:** Hierarchical regression analysis of state anger with demographic variables and coping resources as predictors (N = 169)

Predictor variables	β	*p*	*R* ^2^	*F*
Step 1—demographic variables			0.05	7.561
Culture	0.21	*0.007*		
Step 2—coping resources			0.13	8.89
Culture	0.20	*0.008*		
SOC	−0.29	*0.001*		
Individual hope	−0.18	*0.045*		
Step 3—interaction terms			0.02	0.95

In order to keep the data organized, only variables with significant contribution to the model are presented in the table

*β* standardized regression coefficient, *R* ^2^ proportion of variance explained in each step

### Multiple regression analysis of factors predicting state anxiety

Overall, the regression model explained 25% of the explained variance in state anxiety. As can be seen in Table 5 (below), age significantly predicted state anxiety. Among the coping resources only SOC significantly predicted state anxiety. Higher levels of SOC predicted lower levels of state anxiety. Importantly, the only significant interaction found was that of culture and SOC.

**Table 5:** Hierarchical regression analysis of state anxiety with demographic variables and coping resources as predictors (N = 169)

Predictor variable	β	*p*	*R* ^2^	*F*
Step 1—demographic variables			0.03	3.90
Age	0.19	*0.04*		
Step 2—coping resources			0.18	10.74
SOC	−0.33	*0.000*		
Step 3—interaction terms			0.04	4.11
SOC	−0.43	*0.000*		
Culture × SOC	0.21	*0.007*		

In order to keep the data organized, only variables with significant contribution to the model are presented in the table

*β* standardized regression coefficient; *R* ^2^ proportion of variance explained in each step

To further examine the role of the interaction term SOC × Culture in explaining state anxiety, a bivariate regression analysis was computed for each group with state anxiety was the criterion and SOC was the predictor. The results show that SOC predicted state anxiety only among the Jewish group, but not among the Bedouin Arab group.

## Discussion

The current study explored differences in coping resources and stress reactions among adolescents of two cultural groups as they experienced an escalation in political violence during November of 2012. Specifically, we explored the roles of SOC and hope as predictive factors for the stress reactions of adolescents among two ethnic groups, Israeli Jews and Bedouin Arabs.

Our findings partially support our first hypothesis. The Jewish adolescents reported higher levels of SOC than the Bedouin Arab adolescents. This finding confirms previous research that found that collectivistic minority groups in contexts similar to that of our study exhibit weaker SOC than their individualistic majority counterparts [1]. The results of this study may be related to the lower level of external GRRs among the Bedouin Arab adolescents. Socioeconomic factors such as income and education may be related to the differences in the SOC levels rather than cultural factors related to Bedouin Arab society. Bedouin Arab society is characterized by a lower socioeconomic status and lower levels of education; approximately 40% of the Bedouin Arab population receive welfare services [6, 33, 54, 55].

Contrary to our hypothesis, we found higher levels of both collective hope and individual hope among the Bedouin Arab adolescents. These findings may be explained by the rapid process of modernization within the traditional collectivistic Bedouin Arab population in southern Israel. As members of a highly collectivistic culture, Bedouin individuals are motivated to wish for and promote the goals of others (the collective) before or at the expense of their own personal goals [9, 15, 24, 40, 41]. However, these youths may also be affected by Western individualistic values through the modernization process and those values would encourage them to wish for, expect to achieve, and promote their own personal goals [42].

In terms of stress reactions, we found higher levels of anger among the Bedouin Arab adolescents. This finding may be related to the confusing political situation with which these adolescents were faced. On the one hand, they themselves were living under the threat of missiles from the Gaza Strip, which were falling on their city. On the other hand, these adolescents felt angry with the Israeli leadership that had directed the Israeli military to bomb Gaza. Some of these adolescents had close relatives in the Gaza Strip (Welfare Office, Rahat Municipality, personal communication, June 12, 2014). Moreover, the Bedouin Arab community in southern Israel is also underprivileged in many areas (e.g., social, educational, political, and financial; [33]). All of these conditions may contribute to higher levels of anger and frustration among these adolescents.

Our results showed more complexity concerning the role of SOC in explaining the outcomes among the two cultural groups. A similarity between the groups was observed in the explanation of state anger. SOC levels and individual hope significantly explained state anger among both groups of adolescents; whereas collective hope did not contribute to the explanation of that outcome in either group. An explanation of the latter finding may be related to individual differences within each cultural group. It is not known how strongly those who are identified as Jewish or Bedouin self-identify as such. Our findings regarding the role of SOC are congruent with those of previous studies that reported positive correlations between strong SOC and reduced distress (i.e., [31, 32, 48]). Moreover, our findings regarding the role of individual hope may be explained by previous research concerning the future orientation of adolescents (for a review, see [56]), which indicate that young people all over the globe tend to focus mainly on their own private worlds. It seems that research measures that concern the future (as opposed to the past or present) reveal a more individually oriented perspective and more similarities across different cultural contexts.

However, our results did reveal cultural differences in the context of state anxiety. SOC contributed significantly to the explanation of state anxiety only among the Jewish adolescents. These results underscore the importance of exploring other coping resources and strategies that may play a significant role in explaining state anxiety among Bedouin Arab adolescents. These additional factors may increase the percentage of explained variance of the outcomes (i.e., state anxiety and state anger).

An additional important point is related to gender distribution across the samples. We observed that gender distribution varied widely among our participants (mainly in the Bedouin Arab sample). This unbalanced gender distribution may be explained by the fact that the first author and the two research assistants are females, so that when approached, female adolescents may have felt more encouraged and comfortable to participate and to share these experiences with the research team than males.

Moreover, in term of methodological issues, it is important to address the inconsistency of the responses of Bedouin Arabs to item #10 in the SOC scale. This item was related to the statement “There are many people, even those with strong characters, who sometimes feel miserable\poor”. The inconsistency may be related to the strong tendency among Bedouin Arabs not to disclose their personal problems or their emotions (such as sadness) to others and not exposing their weaknesses to others [11, 57, 58].

### Limitations and directions for future research

This study had some limitations and there are areas that warrant further attention in future research. First, our data were collected in the midst of a war and during a period of missile attacks, thus, some degree of potential sample bias should be taken into account. For example, the samples included a higher percentage of females than males. This bias may reduce the generalizability of the current findings for the general population. Thus, future research should include larger numbers of male and female participants in each cultural group. Moreover, the Bedouin Arab and Jewish participants were approached using different data-collection procedures (in person vs. online, respectively). In future research projects identical data-collection procedures should be used across all cultural groups. Furthermore, our findings were based on self-report measures. Further research using other methods of data collection (e.g., interview techniques, diaries, multi-informant techniques) would be beneficial and important for the evaluation of the validity of the obtained findings. The current study also underscores the need for future research to explore other significant coping resources (e.g., attachment or identification with a group, social support) and coping strategies (i.e., avoidant vs. active) among Bedouin Arab and Jewish adolescents facing escalations in political violence. In addition, a longitudinal study that included both periods of stability and periods of escalated violence would be the best way to examine attitudes, perceptions, and responses among adolescents. Moreover, it would be interesting to evaluate coping resources and stress reactions among parents of these adolescents and test the associations between the results of parents and their offspring.

## Conclusions

The importance of this study lies in its examination of cultural differences and similarities related to coping resources and stress reactions among Bedouin Arab and Jewish adolescents. We found similarities in the ways coping resources predict state anger in different cultural contexts during a period of political violence. SOC and individual hope were significant predictors of state anger in both of the examined cultural groups. However, cultural differences related to coping resources and the relationship between those resources and anxiety were noted. SOC played a significant role in explaining anxiety only among the Jewish adolescents; whereas none of the examined resources predicted state anxiety among the Bedouin Arab adolescents. Our results underscore the importance of examining coping resources and stress reactions among different cultural groups.

### Clinical, educational, and policy implications

Our study compared the coping resources and stress reactions of Bedouin Arab and Jewish adolescents. Such knowledge is expected to help mental-health professionals increase their awareness of the different factors and aspects of emotional distress that characterize each group of adolescents and which resources are important in reducing stress reactions among each cultural group. Bedouin Arab and Jewish adolescents may need similar intervention programs with regard to state anger; whereas different intervention programs may be recommended to help these adolescents manage state anxiety. These programs should be aimed at helping adolescents to cope effectively (by enhancing significant resources) during stressful situations.

## Abbreviations

SOC: sense of coherence
GRRs: generalized resistance resources
HI: hope index
